# A concern about plagiarism

**DOI:** 10.4103/0971-6203.37477

**Published:** 2007

**Authors:** William R. Hendee

**Affiliations:** Editor, Medical Physics; Chair, IOMP Publications Committee, Medical College of Wisconsin, P.O. Box 170970, Whitefish Bay WI 53217. E-mail: whendee@mcw.edu

All scientific journals, including this one, are concerned about plagiarism. The Publications Committee of the International Organization of Medical Physics (IOMP) had prepared an editorial on plagiarism. The editorial is reproduced here (with permission from the IOMP) with slight modifications to enhance its relevance to the audience of this journal.

Plagiarism (from the Latin “*plagiare*”, meaning “to kidnap”) is defined as “the appropriation or imitation of the language, ideas and thoughts of another author and representation of them as one's original work” (*The Random House Dictionary of the English Language* - unabridged). Plagiarism is a serious breach of research ethics that, if committed intentionally, is considered research misconduct. Plagiarism may result in serious sanctions, including public disclosure, loss of research funding, loss of professional stature and termination of employment. Plagiarism undermines the authenticity of research manuscripts and the journals in which they are published and compromises the integrity of the scientific process and the public regard for science. Plagiarism violates the literary rights of individuals who are plagiarized and the property rights of copyright holders. Violation of literary or property rights' may result in legal action against the individual(s) committing plagiarism. Although plagiarism has existed since the beginning of science, it seems to be increasing because the World Wide Web (Internet) facilitates finding and copying the work of others

It is possible not only to plagiarize the works of others, but also one's own work through reuse of identical or nearly identical portions of manuscripts without acknowledgement or citation. Simultaneous or subsequent submission of similar manuscripts with only minor differences and without citation between the manuscripts is, unfortunately, a rather common practice among authors hoping to acquire multiple publications from a research project. Scientific journals discourage this practice, and usually will not permit it if exposed before publication. Occasionally, the same (or a very similar) article may be published in two journals, because the journals reach different audiences and the article is of interest to both. This practice must be approved by the editors of both journals, and the duplication must be acknowledged in each article

When there is a possibility of plagiarism (often through an allegation of plagiarism by the original author, a reviewer or an interested third party), the journal's editor should act quickly. The editor should examine the original material and the publication alleged to constitute plagiarism. If the editor concludes that no plagiarism has occurred, the accuser should be notified, and no further action is necessary. If the evidence suggests that plagiarism may have occurred, the editor should contact the accused author(s), the author(s) whose work may have been plagiarized and the copyright holder of the original material if different from the author(s). The correspondence should include the alleged plagiarizing language and a copy of the original and suspected work. If all parties agree that plagiarism (whether intentional or unintentional) has occurred, a written letter of apology should be sent promptly by the offending author(s) to the editor and to the author(s) and copyright holder whose work has been plagiarized. If the offending work has been published, a notice of plagiarism, citing both the plagiarized and the offending articles and containing the exact text that has been plagiarized, should be published in the next available article of the journal in which the offending article was published. The plagiarizing authors must agree that all dissemination of the offending article will to be accompanied by the notice of plagiarism

If the accused author(s) deny that plagiarism has occurred, the editor must explore the accusation further, preferably through a mechanism already established by the journal to investigate allegations of scientific misconduct. All parties to the allegation should be encouraged to submit corroborating evidence, and the accused author(s) should be granted an opportunity (at no expense to the journal) to testify in person in defense against the allegation. The investigation should be concluded within a reasonable period of time (e.g. 3 months)

If the mechanism to investigate the allegation of plagiarism concludes in support of the allegation, then the process for the case in which plagiarism is admitted should be instituted. Further, the editor must decide whether the plagiarism should be reported to the guilty parties' supervisor, employer and/or professional organization. If the mechanism rules against the accusation of plagiarism, a letter stating this ruling should be provided to the accuser, the author(s) accused of plagiarism, the author(s) of the original work and the copyright holder if different from the author(s). In either case, these actions should constitute closure of the allegation of plagiarism

An allegation of plagiarism is a serious accusation and should never be taken lightly. On the other hand, self-policing is a major strength of the scientific community, and plagiarism should always be reported when it is suspected to have occurred

